# Nasosorption as a Minimally Invasive Sampling Procedure: Mucosal Viral Load and Inflammation in Primary RSV Bronchiolitis

**DOI:** 10.1093/infdis/jix150

**Published:** 2017-03-27

**Authors:** Ryan S. Thwaites, Kazuhiro Ito, Jasmine M. S. Chingono, Matthew Coates, Hannah C. Jarvis, Tanushree Tunstall, Lauren Anderson-Dring, Lindsey Cass, Garth Rapeport, Peter J. Openshaw, Simon Nadel, Trevor T. Hansel

**Affiliations:** 1 National Heart and Lung Institute, Faculty of Medicine, Imperial College London,; 2 Pulmocide Ltd, and; 3 Paediatric Intensive Care Unit, St Mary’s Hospital, Imperial College Healthcare Trust, London, United Kingdom

**Keywords:** respiratory syncytial virus, nasosorption, viral load, bronchiolitis, respiratory sampling.

## Abstract

**Background.:**

Existing respiratory mucosal sampling methods are flawed, particularly in a pediatric bronchiolitis setting.

**Methods.:**

Twenty-four infants with bronchiolitis were recruited: 12 were respiratory syncytial virus (RSV)–positive, 12 were RSV-negative. Infants were sampled by nasosorption and nasopharyngeal aspiration (NPA).

**Results.:**

Nasosorption was well tolerated and identified all RSV+ samples. RSV load measured by nasosorption (but not NPA) correlated with length of hospital stay (*P* = .04) and requirement for mechanical ventilation (*P* = .03). Nasosorption (but not NPA) levels of interferon γ, interleukin 1β, CCL5/RANTES, and interleukin 10 (IL-10) were elevated in RSV+ bronchiolitis (all *P* < .05), furthermore CCL5 and IL-10 correlated with RSV load (*P <* .05).

**Conclusions.:**

Nasosorption allowed measurement of RSV load and the mucosal inflammatory response in infants.

## Summary

We used nasosorption as a noninvasive method of respiratory mucosal sampling in a paediatric virology setting. Nasosorption was well tolerated and demonstrated respiratory syncytial virus load to be associated with disease severity and elevated mediators of inflammation.

Respiratory syncytial virus (RSV) bronchiolitis of infancy is a major global cause of morbidity and mortality [1]. Nasal samples are used for clinical viral diagnostics as well as research measurements of viral load and the immune response. The standard method for diagnosis of RSV infection uses nasopharyngeal aspiration (NPA) to obtain samples, but this is an invasive and unpleasant procedure for the child, making it difficult to repeat sampling. Nasopharyngeal aspiration may also be inaccurate as a research tool, causing variable dilution of virus and mediators [2]. These flaws limit NPA’s utility as a research tool. Cotton, rayon, and synthetic flocked nasal swabs have also been used, but studies have observed variable sensitivity and reliability for determination of respiratory viral infections, including RSV [3–5]. Additionally, these swabs can bind proteins nonspecifically, limiting their use for measuring mucosal inflammatory mediators. Nasal swabs also require rotation within the nose, which is uncomfortable for the infant [4].

Nasosorption (NS) is a noninvasive alternative method to sample nasal mucosal lining fluid using a synthetic absorptive matrix (SAM) of low protein binding. The SAM is advanced up the lumen of the nasal cavity, and then the outside of the nose is gently pressed to oppose the SAM against the nasal mucosa for 30 seconds. The SAM is not rotated in the nostril. Nasosorption has been used to measure cytokine levels in infants [6] and following nasal allergen challenge in adults [7]. We aimed to assess the utility of NS for the determination of RSV infection, viral load, and the mucosal immune response in infants with viral bronchiolitis.

## METHODS

### Patients and Sampling

Informed consent was given by parents or guardians of infants before recruitment. Nasosorption and NPA sampling were conducted on infants aged between 4 weeks and 1 year admitted to St Mary’s Hospital, Paddington, London, United Kingom, over winter 2015–2016. Infants with any known preexisting respiratory condition, a corrected gestational age <34 weeks, or a weight <3 kg were excluded from the study. The definition of bronchiolitis for inclusion was based on standard clinical criteria (cough, tachypnoea, wheeze, crackles and wheezes on auscultation, and hyperinflation). Requirement for mechanical ventilation was used to stratify RSV+ patients as those that required ventilation at any point in their in-patient stay (pediatric intensive care unit [PICU]) or those that did not (Wards). Nasosorption was performed by manipulating the swab up the nostril lumen, avoiding brushing of the mucosa, and then making mucosal contact for 30 seconds by the clinician using light finger pressure from outside the nose. We used CE-marked NS medical devices (Hunt Developments UK Ltd) [2]. Nasosorption was repeated following a 1-minute interval in either the same naris (PICU cases) or once in each naris (healthy control subjects and Wards). Following NS, NPA was performed by direct aspiration, followed by flushing the suction tubing with 3.5 mL of saline to remove secretions. All samples were immediately stored on ice and processed within 3 hours, prior to storage at −80°C.

### Laboratory Experiments

Viral load was measured by quantitative reverse transcription polymerase chain reaction (qRT-PCR) using MagMax 96 viral RNA isolation kits (ThermoFisher) and Precision OneStep qRT-PCR kits for RSV-A and RSV-B (PrimerDesign). The immune mediators interferon γ (IFN-γ), C–X–C motif chemokine 10 (CXCL10/IP10), interleukin 10 (IL-10), interleukin 12p70, interleukin 13 (IL-13), interleukin 1β (IL-1β), interleukin 2, interleukin 4 (IL-4), interleukin 5 (IL-5), interleukin 6, CXCL8, tumour necrosis factor α (TNF-α), C–C motif chemokine 5 (CCL5/RANTES), interferon α2a, and granulocyte macrophage colony-stimulating factor (GM-CSF) were measured by multiplex immunoassay (Mesoscale Discovery). Mediator data are expressed as raw values in the figures using a log10 scale.

### Statistical Tests

Statistical tests were performed using GraphPad Prism version 7 (GraphPad Software). Categorical demographics data were analyzed using Fisher’s exact test; continuous demographics data were analyzed using 2-tailed unpaired *t* tests or Mann–Whitney *U* tests as appropriate. Nasosorption cytokine data were analzsed using Kruskall–Wallis tests with Dunn’s correction for multiple comparisons, 2-tailed Mann–Whitney *U* tests were used for NPA cytokine data, and correlations were assessed using 2-tailed Spearman tests. As an exploratory trial of NS in pediatric RSV bronchiolitis, no power calculations were performed before study recruitment.

### Ethical Approval

The study was approved by the Black Country Research Ethics Committee (REC no. 15/WM/0343).

## RESULTS

Twelve infants with bronchiolitis were positive for RSV infection, as determined by clinical respiratory viral screening of NPA samples. Patient demographics are summarized in Supplementary Table 1. No significant demographical differences were observed between RSV+ and RSV− groups. However, there was a trend toward younger age, male sex, and increased requirement for mechanical ventilation in the RSV+ group, as reported elsewhere [8]. Nine healthy control subjects were recruited; 5 were boys, and the mean age was 180 days (range = 33–313).

**Figure 1.**
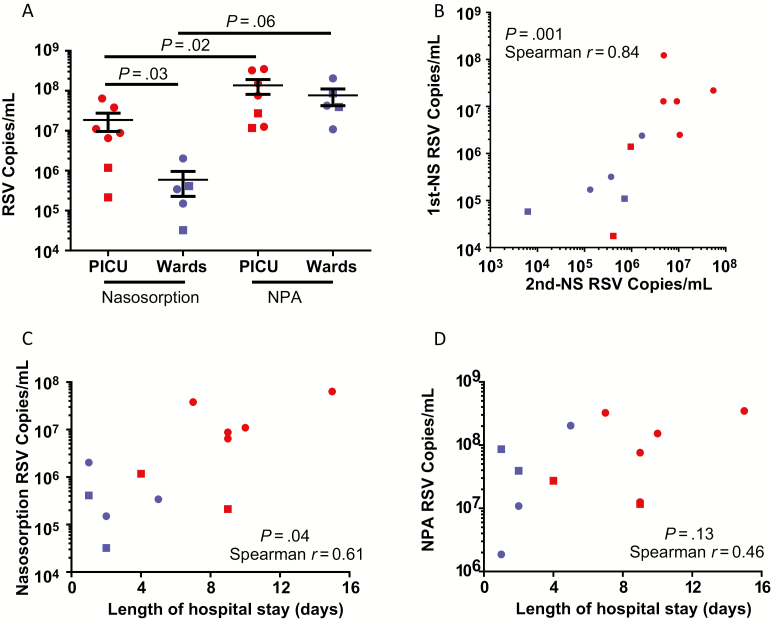
Nasosorption (NS) and nasopharyngeal aspiration (NPA) for respiratory syncytial virus (RSV) load quantitation. *A*, RSV load measured by NS and NPA from infants admitted to the pediatric intensive care unit (PICU) on mechanical ventilation (n = 7) and nonventilated infants on general paediatric wards (n = 5). *B*, Reproducibility of NS RSV load measurements (n = 12; same subjects and data as *A*, see Methods). Correlation between length of admission and RSV load as measured by NS (*C*) and NPA (*D*). RSV-A, circle; RSV-B, square; RSV+ Wards/nonventilated, blue; RSV+ PICU/ventilated, red. Data were analyzed by 2-tailed Mann–Whitney *U* tests, 2-tailed Wilcoxon matched pairs tests, or Spearman correlations as applicable. Abbreviations: NPA, nasopharyngeal aspiration; NS, nasosorption; PICU, pediatric intensive care unit; RSV, respiratory syncytial virus.

All 12 infants positive for RSV infection by clinical viral screening were also RSV+ when assessed by RT-qPCR of both NPA and NS samples. No infants who were RSV− by clinical viral screening were RSV+ by RT-qPCR. Despite this diagnostic equivalence, viral loads were several-fold lower in NS than NPA, as summarized in Supplementary Table 2. This difference was significant in PICU (n = 7) cases (*P* = .02) (Figure 1A). A similar but nonsignificant trend was observed in the smaller number of subjects in the Ward patients group (n = 5; *P* = .06) (Figure 1A). Respiratory syncytial virus load by NS (but not NPA) was significantly lower in Ward patients relative to PICU cases (*P* = .03) (Figure 1A). Moreover, a significant correlation was observed between viral load measurements on repeated NS (Figure 1B), whereas repeated NPA was declined by parents. Bland-Altman analysis of viral load data showed good equality between repeated NS samples, although samples with very high or very low viral load had poorer reproducibility (Supplementary Figure 1). A significant correlation between RSV load and length of hospital stay (a measure of disease severity [9, 10]) was observed when measured by NS (Figure 1C), which was not evident with NPA (Figure 1D). This correlation was also evident on NS when only RSV-A cases were considered (n = 8; *P* = .03; Spearman *r* = 0.78; data not shown).

**Figure 2.**
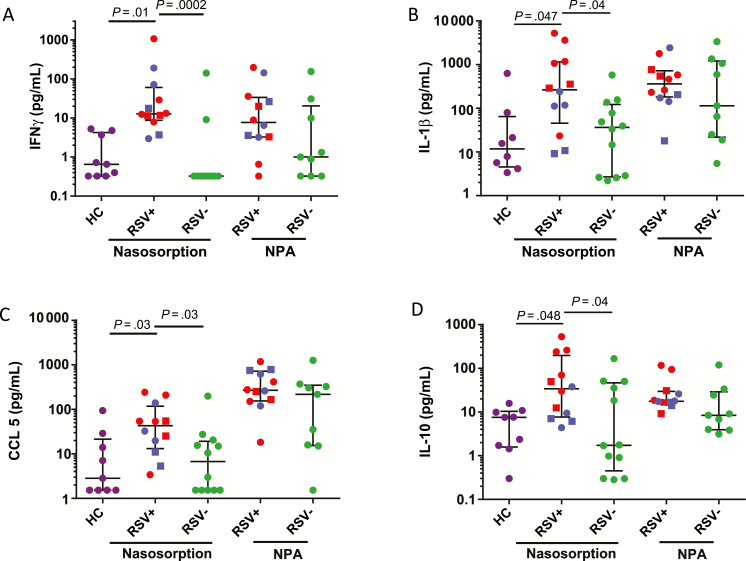
Nasosorption (NS) associates respiratory syncytial virus (RSV) infection with elevated mucosal cytokines. Levels of interferon γ (*A*), interleukin 1β (*B*), CCL5/RANTES (*C*), and interleukin 10 (*D*) as measured by NS from healthy control subjects (HC, n = 9) as well as NS and nasopharyngeal aspiration (NPA) from bronchiolitic patients associated with RSV (RSV+, n = 12) and other respiratory infections (RSV−, n = 12). RSV-A, circle; RSV-B, square; RSV+ Wards/nonventilated, blue; RSV+ PICU/ventilated, red. Nasosorption cytokine data were analyzed using Kruskall–Wallis test with Dunns correction for multiple comparisons, 2-tailed Mann–Whitney *U* tests were used for NPA cytokine data. Abbreviations: HC, healthy control; IFN-γ, interferon γ; IL-1β, interleukin 1β; IL-10, interleukin 10; NPA, nasopharyngeal aspiration; RSV, respiratory syncytial virus.

Measurements by NS demonstrated that relative to both RSV− bronchiolitis and healthy control subjects, RSV+ bronchiolitis was associated with higher levels of IFN-γ, IL-1β, CCL5/RANTES, and IL-10 (Figure 2A–D, respectively) (*P* < .05 throughout). No significant differences were observed in measurements of these cytokines from NPA samples. Additionally, no significant differences were observed between groups in the levels of other measured mediators, including IL-4, IL-5, and IL-13, in any sample (data not shown). Levels of inflammatory mediators were reproducible between nares of an individual or following repeated NS sampling of the same naris at a 1-minute interval (data not shown). Measurements of viral load and mediator levels did not correlate between matched NPA and NS samples, suggesting that these samples are inherently different (data not shown).

When measured by NS, viral load significantly positively correlated with CCL5 (Supplementary Figure 2A) and IL-10 (Supplementary Figure 2B). By comparison, no significant association was observed between RSV load and any mediator when measured by NPA (data not shown).

## Discussion

This exploratory study found NS to be a useful sampling method for viral diagnostics, measurement of RSV load, and assessment of mucosal cytokine levels in babies hospitalized with bronchiolitis. Nasosorption was well tolerated by babies, parents, and nursing staff in this study, although no objective measures of tolerability were performed.

Nasosorption is performed using a SAM of low protein binding and high absorption (wicking capacity). In contrast, swabs containing plant-derived materials such as cotton or rayon may bind proteins to a variable extent, resulting in difficulties in sample elution. Nasopharyngeal aspiration is the standard upper respiratory sampling technique for the assessment of RSV load in infants [9, 11]. Extensive efforts have been made to standardize the NPA technique to attempt equivalent dilution of samples. Either saline is instilled into the naris of the child before suction or the sample is first aspirated and then flushed with saline through the NPA catheter [9, 11]. However, these NPA techniques result in unknown and variable dilution of mucosal secretions and are difficult to repeat. By contrast, in our study, NS measurements gave tightly correlated data in viral load and cytokine measurements. Due to mechanical ventilation through 1 nostril in our PICU cases, the opposing nostril was sampled twice at a 1-minute interval. In comparison, Wards cases were sampled once in each nostril. Both techniques showed good repeatability; however, this approach prevents direct comparisons between repeatability of PICU and Wards data.

Apart from a possible clinical application of NS sampling for diagnostic purposes in bronchiolitis, reliable biomarker endpoints are needed for the assessment of new molecular entities, monoclonal antibodies, and vaccines in clinical trials [12]. In particular, because NS was so well tolerated, it can be repeated frequently, unlike NPA. This creates the potential to have greater utility in the kinetic assessment of viral load and the immune response and to involve fewer infants to establish a therapeutic effect.

We observed an excess of male patients of younger age and with the need for mechanical ventilation in the RSV+ group relative to the RSV− group, in accordance with other studies [8]. Despite the small sample size of this study, viral load measured by NS correlated with the length of hospital stay of RSV-infected infants, supporting previous reports using NPA in larger cohorts [9, 10]. The low RSV loads observed by NS, relative to NPA, could limit sensitivity in cases of low viral load. This difference between NS and NPA could be due to incomplete elution of virus from the NS matrix, a possibility that is currently under investigation. In the future it will be important to assess whether elution of nasosorption samples using an RNA lysis buffer would enhance viral yields. Additionally, coinfection of RSV cases with other viruses or pathogenic bacterial species were not assessed and could influence the results. We found that requirement for mechanical ventilation was associated with higher viral loads on NS, an observation supported by experimental RSV infection of adults, in which higher viral load was associated with greater disease severity [13]. In addition, we observed a correlation between viral load and the infant’s immune response in terms of nasal cytokines. Interestingly, RSV infection was associated with elevated levels of the inflammatory mediators CCL5, IL-10, IL-1β, and IFN-γ, which are predominantly involved in the type I antiviral inflammatory response. These mediators have been associated with RSV infection in some other studies, although, interestingly, this study did not observe type II inflammation (IL-4, IL-5, and IL-13) to be associated with RSV infection [14, 15].

This study therefore supports the concept of viral load as a critical determinant of RSV pathogenesis. However, these findings should be validated in larger cohorts and at multiple study sites, and further validation of NS versus swabs and NPA sampling should be conducted. In conclusion, we report NS to be a promising, noninvasive method for pediatric respiratory sampling that may be an alternative, or complementary, approach to NPA.

## Supplementary Data

Supplementary materials are available at *The Journal of Infectious Diseases* online. Consisting of data provided by the authors to benefit the reader, the posted materials are not copyedited and are the sole responsibility of the authors, so questions or comments should be addressed to the corresponding author.

## Supplementary Material

Supplementary_Figure_1

Supplementary_Figure_2

Supplementary_Table_1

Supplementary_Table_2

Supplementary_Figure_Table_Legends

## Notes

***Acknowledgments.*** T. T. H. and S. N. were responsible for study concept and design. R. T., K. I., M. C., and H. A. acquired the data. J. C., L. M., H. J., and S. N. collected study samples. R. T., K. I., T. T., and T. T. H. analyzed and interpreted the data. R. T. and T. T. H. drafted the manuscript, which was critically revised for important intellectual content and approved by all authors. R. T. is guarantor.

***Disclaimer.***  The views expressed are those of the authors and not necessarily those of the National Health Service, the National Institue for Health Research, the Department of Health, or Public Health England.

***Financial support.*** This work was supported by funding from the Imperial National Institute for Health Research (NIHR) Biomedical Research Centre, the NIHR Health Protection Research Unit in Respiratory Infections at Imperial College London in partnership with Public Health England, and the NIHR Imperial Patient Safety Translational Research Centre and the Respiratory Syncytial Virus Consortium in Europe. Pulmocide Ltd provided additional funding.

***Potential conflicts of interest.*** All authors: No reported conflicts of interest. All authors have submitted the ICMJE Form for Disclosure of Potential Conflicts of Interest. Conflicts that the editors consider relevant to the content of the manuscript have been disclosed.
