# Supplementary figures and images for: Nasosorption as a Minimally Invasive Sampling Procedure: Mucosal Viral Load and Inflammation in Primary RSV Bronchiolitis
Source: J Infect Dis. 2017 Mar 27;215(8):1240–4. doi: 10.1093/infdis/jix150 (PMC5441107; doi:10.1093/infdis/jix150)

## Slide 1
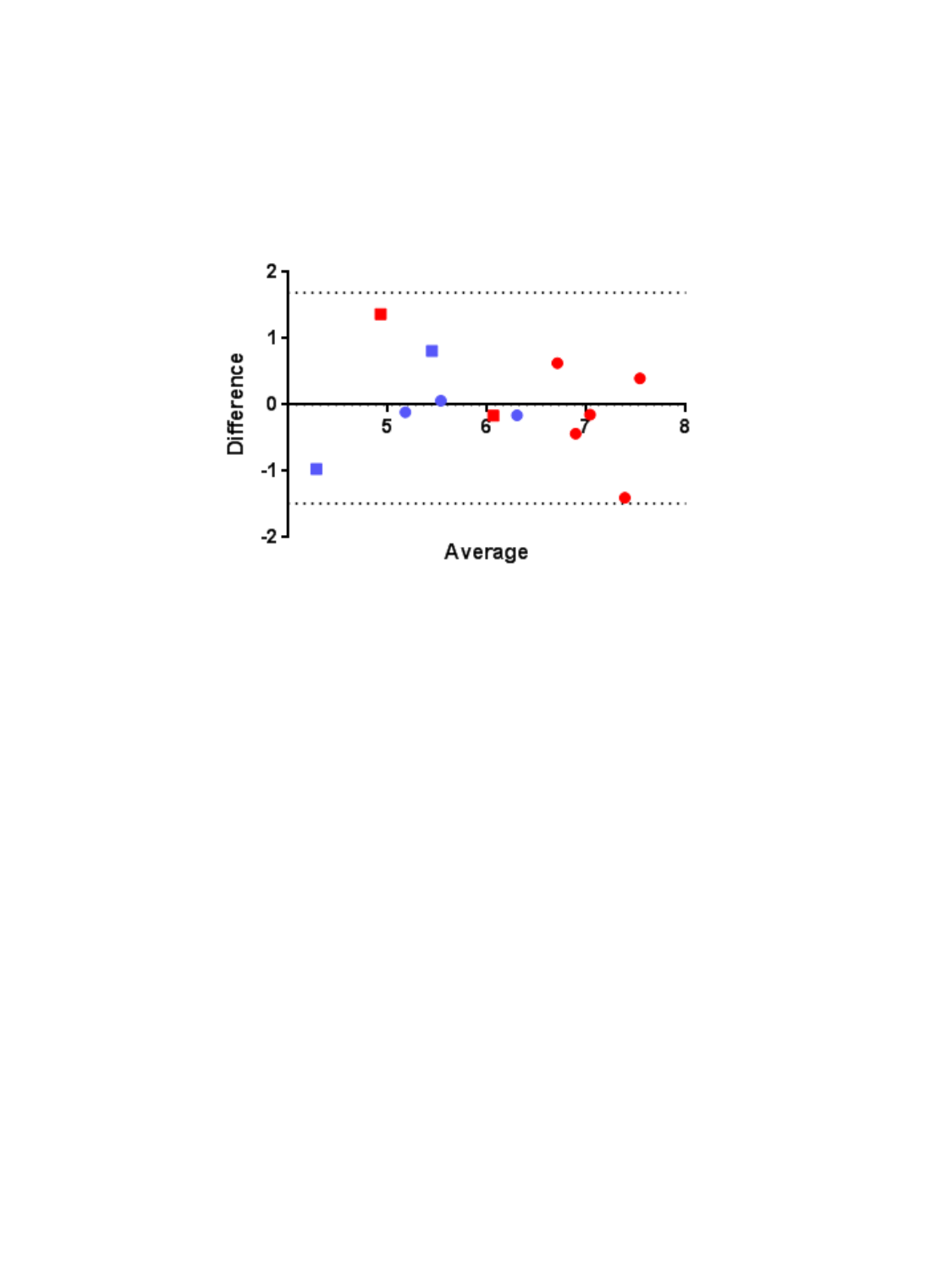

Supplement: Supplementary_Figure_1 [file jix150_suppl_Supplementary_Figure_1.pptx]

## Slide 1
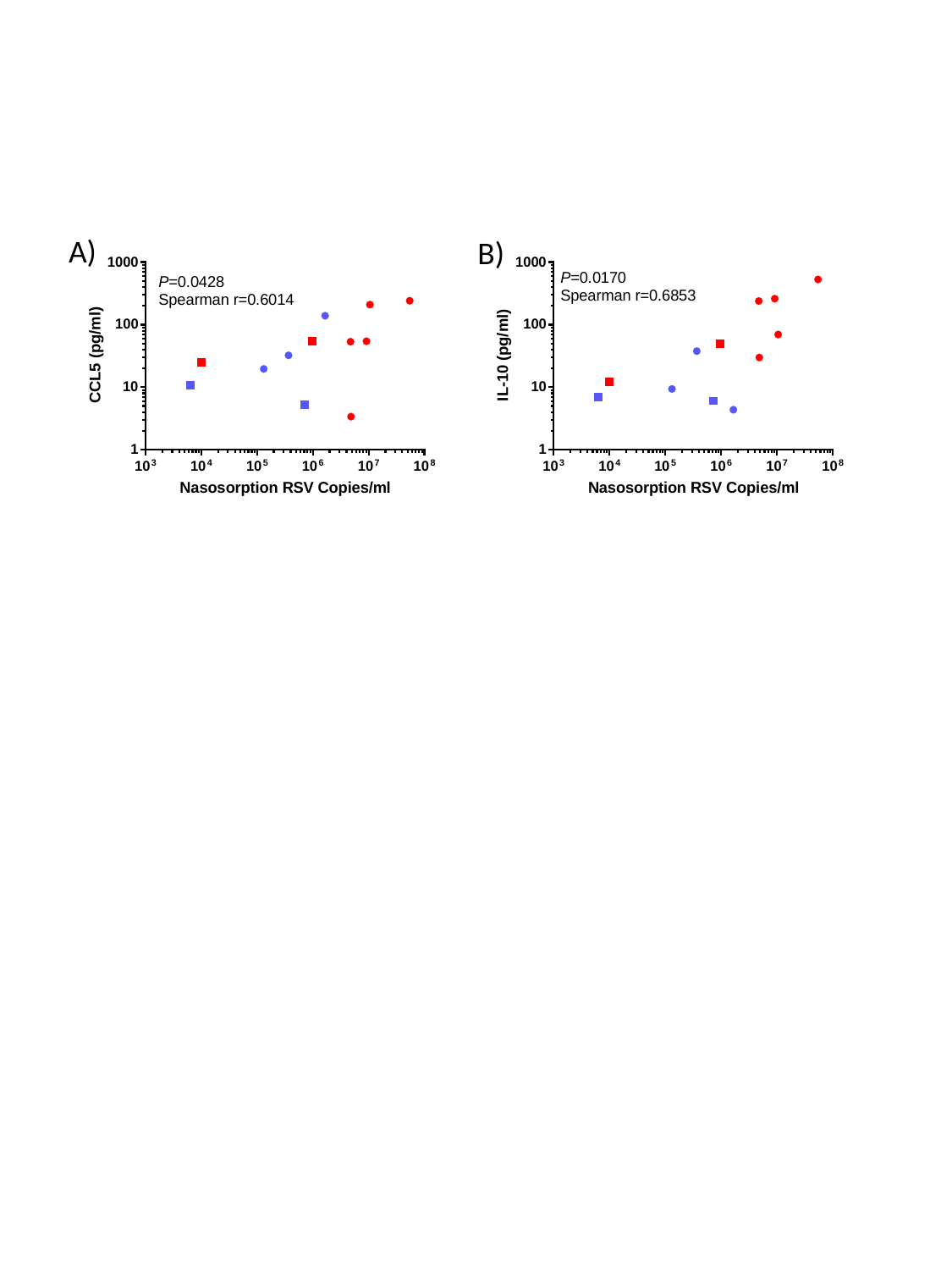

A)
B)

Supplement: Supplementary_Figure_2 [file jix150_suppl_Supplementary_Figure_2.pptx]
