# Supplementary material for: Nasosorption as a Minimally Invasive Sampling Procedure: Mucosal Viral Load and Inflammation in Primary RSV Bronchiolitis
Source: J Infect Dis. 2017 Mar 27;215(8):1240–4. doi: 10.1093/infdis/jix150 (PMC5441107; doi:10.1093/infdis/jix150)
Supplement: Supplementary_Table_1 [file jix150_suppl_Supplementary_Table_1.docx]

|  | **RSV+ bronchiolitis (n=12)** | **RSV- bronchiolitis (n=12)^†^** | ***P*-value** |
| --- | --- | --- | --- |
| Male gender (% of group) | 10 (83%) | 6 (50%) | 0.193 |
| Age (days, mean) [range] | 84  [29 – 205] | 187  [40 – 365] | 0.075 |
| Prematurity | 5/12 (42%) | 2/12 (17%) | 0.371 |
| Comorbidity | 4/12 (33%) | 4/12 (33%) | 0.640 |
| Admission to PICU & Mechanical ventilation | 7/12 (58%) | 2/12 (17%) | 0.089 |
| Symptom onset to sampling (days, mean) [range] | 3.4 [1-7] | 2.5 [0-7] | 0.240 |
| Length of hospital stay (days, median) [range] | 4 [0-15] | 2 [0-42] | 0.106 |
| RSV type (A:B) | 8:4 | - | - |
