# Supplementary material for: Nasosorption as a Minimally Invasive Sampling Procedure: Mucosal Viral Load and Inflammation in Primary RSV Bronchiolitis
Source: J Infect Dis. 2017 Mar 27;215(8):1240–4. doi: 10.1093/infdis/jix150 (PMC5441107; doi:10.1093/infdis/jix150)
Supplement: Supplementary_Table_2 [file jix150_suppl_Supplementary_Table_2.docx]

|  | **Nasosorption PICU (n=7)** | **Nasosorption Wards (n=5)** | **NPA PICU (n=7)** | **NPA Wards (n=5)** |
| --- | --- | --- | --- | --- |
| Median | 8.82x10^6^ | 3.43x10^5^ | 7.61x10^7^ | 4.22x10^7^ |
| Lower 95% confidence interval of median | 2.14x10^5^ | 3.23x10^4^ | 1.17x10^7^ | 1.08x10^7^ |
| Upper 95% confidence interval of median | 6.37x10^7^ | 2.03x10^6^ | 3.49x10^8^ | 2.04x10^8^ |
