# Supplementary material for: Nasosorption as a Minimally Invasive Sampling Procedure: Mucosal Viral Load and Inflammation in Primary RSV Bronchiolitis
Source: J Infect Dis. 2017 Mar 27;215(8):1240–4. doi: 10.1093/infdis/jix150 (PMC5441107; doi:10.1093/infdis/jix150)
Supplement: Supplementary_Figure_Table_Legends [file jix150_suppl_Supplementary_Figure_Table_Legends.docx]

**Supplementary Figure 1. Bland-Altman plot of nasosorption RSV load repeatability.** Log-transformed repeatability data from matched nasosorption samples. *RSV-A, circle; RSV-B, square; RSV+ Wards/Non-ventilated, blue; RSV+ PICU/Ventilated, red. 95% confidence intervals shown as dotted lines.*

**Supplementary Figure 2. High RSV load correlates with elevated inflammatory mediators.** Correlation between RSV viral load, as assessed by nasosorption, and A) CCL5 and B) IL-10 in bronchiolitic patients (n=12). *RSV-A, circle; RSV-B, square; RSV+ Wards/Non-ventilated, blue; RSV+ PICU/Ventilated, red.*

**Supplementary Table 1. Clinical characteristics of bronchiolitis cases**

Categorical data were analysed using Fishers Exact test; continuous data were analysed using two tailed Unpaired t-tests or Mann-Whitney U-tests as appropriate. **^†^** (6 rhinovirus, 1 metapneumovirus, 1 parainfluenza and 4 with no identified pathogen).

**Supplementary Table S2 – Viral load is nasosorption and NPA samples**
